# Adsorption of Norfloxacin on Urban River Sediments: Site Energy Distribution and Effects of Coexisting Ions

**DOI:** 10.3390/molecules31213997

**Published:** 2026-11-06

**Authors:** Shiyong Tao, Tianfang Wei, Luyao Jin, Miaotong Jiang, Yilin Chen, Jing Xu

**Affiliations:** 1 State Key Laboratory of Water Resources Engineering and Management, Wuhan University, Wuhan 430072, China; taoshiyong@whu.edu.cn (S.T.); weitfang@whu.edu.cn (T.W.); luyaojin@whu.edu.cn (L.J.); jiangmiaotong@whu.edu.cn (M.J.); yilinchen0817@whu.edu.cn (Y.C.); 2 Hubei Key Laboratory of Water System Science for Sponge City Construction, Wuhan University, Wuhan 430072, China

**Keywords:** fluoroquinolone antibiotics, sediments, adsorption, site energy distribution, coexisting ions

## Abstract

The environmental fate of fluoroquinolone antibiotics in urban rivers is controlled by sediment characteristics and water chemistry, yet how these factors modulate adsorption site energetics remains insufficiently quantified. In this study, norfloxacin (NOR) adsorption on urban river sediments from Wuhan, China, was investigated using adsorption isotherms and site energy distribution analysis. All sediments followed the Langmuir model (R^2^ = 0.981–0.998) with saturated adsorption capacities of 17.932–35.476 mg/g, indicating pronounced sediment-dependent retentions. Site energy distribution analysis revealed clear differences in both characteristic adsorption energy E_0_^*^ and the peak of frequency distribution curves F(E_0_^*^) between the sediments. Higher adsorption capacities were found not necessarily linked to higher adsorption energies, but rather to higher frequencies of effective adsorption sites. Coexisting ions exerted ion-specific effects on NOR adsorption. Common electrolytes suppressed adsorption and shifted site energy distributions toward lower-energy regions, with Na_2_SO_4_ exerting a stronger effect than NaCl due to its higher ionic strength. Ca^2+^ had little effect, whereas Mg^2+^ slightly enhanced adsorption. Fe^3+^ caused a slight enhancement, while Cu^2+^ markedly increased NOR adsorption and shifted the site energy distributions toward higher-energy regions. These findings demonstrate that site energy distribution analysis provides a useful framework for interpreting ion-modulated antibiotic adsorption on urban river sediments.

## 1. Introduction

The widespread presence of antibiotics in aquatic environments and the associated ecological risks have become a global environmental issue. Continuous discharges from municipal wastewater, medical facilities, and urban runoff have led to frequent detection of antibiotics in surface water, raising increasing concerns regarding their environmental persistence and potential biological impacts [1,2]. In aquatic systems, sediments constitute a crucial environmental interface and play a dual role. On the one hand, sediments act as sinks for antibiotics by temporarily immobilizing them through adsorption. On the other hand, changes in environmental conditions may trigger their remobilization, turning the sediments into secondary sources that regulate the transport, fate, and bioavailability of contaminants [3,4]. Hence, a comprehensive understanding of antibiotic adsorption onto sediments is essential and urgent for robust ecological environment assessments.

The characteristics of urban river sediments exhibit significant spatial variability, thus often yielding distinct adsorption behaviors for a given contaminant within the urban watershed [5]. In addition, natural waters contain a wide range of coexisting inorganic ions, including water chemistry ions (e.g., Na^+^, Cl^−^, SO_4_^2−^, Ca^2+^, and Mg^2+^) and metal ions (e.g., Fe^3+^ and Cu^2+^) [6,7]. These ions can modulate antibiotic adsorption through multiple pathways, such as competition for surface adsorption sites, alteration of sediment surface charge, or formation of soluble or surface-bound complexes [8,9,10]. Overall, antibiotic adsorption in real aquatic environments is influenced by the combination of sediment characteristics and complex solution chemistry, considerably complicating the prediction of their environmental fate [11,12].

Antibiotic adsorption studies have mainly relied on empirical isotherm models, most notably the Langmuir model, to describe the macroscopic adsorption behaviors and compare adsorption capacities among different solid substrates [13]. Although this model contributes to the quantitative characterization, it is inherently based on simplified assumptions about the homogeneity of adsorption sites. In natural sediments, however, the coexistence of organic matter, clay minerals, and metal oxides generates a wide spectrum of adsorption sites with distinct binding energies [14,15,16]. Under such surface conditions, especially at environmentally relevant low solute concentrations, the applicability of the Langmuir model is often compromised, accompanied by increased fitting uncertainty. That is to say, the conventional isotherm parameters alone are insufficient to address the intrinsic energetic heterogeneity of antibiotic adsorption, limiting mechanistic interpretation at the microscopic level.

In recent years, the adsorption site energy distribution theory (SEDT) has been increasingly adopted to characterize the heterogeneous adsorption systems involving organic contaminants and heavy metals [17,18,19,20]. By transforming macroscopic adsorption isotherms into continuous energy distribution curves, this approach enables the identification of adsorption sites with different binding strengths, providing direct insights into the non-uniform nature of adsorbent surfaces [21]. However, its application to antibiotic adsorption on compositionally complex natural river sediments remains limited, especially in studies involving the influence of coexisting ions.

Among various classes of antibiotics, fluoroquinolones have received particular environmental attention, given their extensive usage, broad-spectrum antibacterial activity, and strong affinity for solid phases [22,23]. In this study, the adsorption of norfloxacin (NOR), a representative fluoroquinolone antibiotic, on urban river sediments was investigated through batch adsorption experiments to elucidate how sediment characteristics and coexisting ions regulate its retention behaviors. Adsorption site energy distribution analysis was applied to resolve the energetic heterogeneity and relative contribution of adsorption sites. The effects of common coexisting ions were further examined to reveal the ion-specific regulation of NOR adsorption and site energy distributions. By integrating conventional isotherm modeling with energy-based analysis, this work provides mechanistic insights into NOR retention on urban river sediments and contributes to improving the assessment of antibiotic persistence in urban aquatic environments.

## 2. Results and Discussion

### 2.1. Adsorption Isotherms of NOR on Different Sediments

**Figure 1:**
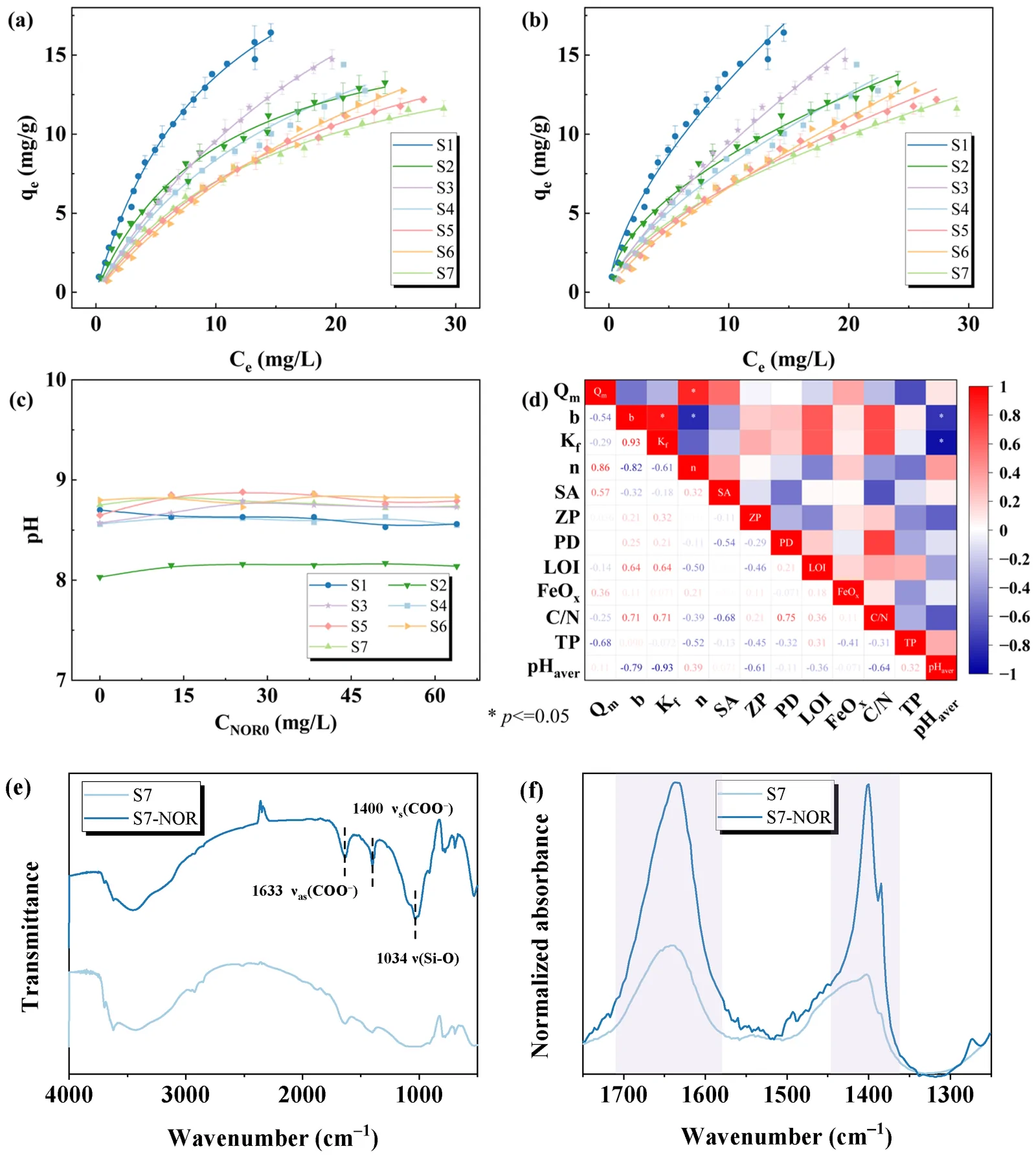
Adsorption isotherms of NOR on sediments fitted with the (**a**) Langmuir and (**b**) Freundlich models, respectively; (**c**) final equilibrium pH across different initial NOR concentrations; (**d**) correlation matrix between isotherm parameters and sediment physicochemical characteristics; (**e**) full transmittance spectra of S7 and S7-NOR; (**f**) enlarged absorbance spectra in the 1750–1250 cm^−1^ region, showing the characteristic peaks of NOR. Experimental conditions: sediment dosage = 3 g/L; [NOR]_0_ = 10–200 μM for isotherm adsorption; [NOR]_0_ = 100 μM for FTIR analysis.

The adsorption isotherms of NOR on the collected urban river sediments are shown in Figure 1a,b. Due to the strong buffering capacity of the sediments, the final equilibrium pH of the solutions was governed by the intrinsic properties of each sediment. Consequently, the final pH differed among different sediments under identical initial NOR concentrations but remained essentially constant for a given sediment across the entire concentration range tested (Figure 1c). For all sediments, the equilibrium adsorption amounts (q_e_) increased with increasing equilibrium concentrations (C_e_), while the rate of increase gradually decreased at higher concentrations, indicating progressive occupation of available adsorption sites and an approach toward saturation. Although the overall isotherm shapes were similar, clear differences in adsorption capacity were observed among sediments, suggesting sediment-dependent NOR retention behaviors under identical experimental conditions.

Both the Langmuir (Figure 1a) and Freundlich (Figure 1b) models were fitted and compared using R^2^ and RMSE. The fitting parameters are summarized in Table 1. The Langmuir model provided a better description of the experimental data, yielding higher R^2^ values (0.981–0.998) and lower RMSE (0.172–0.541 mg/g) than the Freundlich model. Langmuir was therefore selected, indicating that NOR adsorption could be reasonably approximated by a finite site adsorption process. However, this apparent agreement should not be interpreted as evidence for truly homogeneous adsorption surfaces, as natural sediments contain multiple reactive components and heterogeneous binding domains [11,12,16]. The Langmuir-derived saturated adsorption capacity (Q_m_) varied from 17.932 to 35.476 mg/g among the urban river sediments. This approximately two-fold difference suggests that NOR retention was influenced by the abundance and accessibility of effective adsorption sites in the sediments.

To further explore the potential influence of sediment characteristics on adsorption behaviors, correlation analysis was conducted among isotherm parameters and selected physicochemical characteristics, including the BET surface area (SA), loss on ignition (LOI), iron oxides (FeO_x_) content, zeta potential (ZP), particle diameter (PD), carbon-to-nitrogen ratio (C/N), total phosphorus (TP) content, and the average equilibrium pH (pH_aver_), as shown in Figure 1d. Given the limited number of sediment samples (n = 7), this analysis has low statistical power, and the correlation results are thus presented as exploratory trends only. The exploratory analysis revealed that Q_m_ showed a moderate positive correlation with SA (r = 0.57), which is consistent with the general expectation that larger surface areas provide more interfacial domains for interactions [15,24]. Q_m_ also showed a weak positive trend with FeO_x_ content (r = 0.36). FeO_x_ can strongly influence antibiotic adsorption through surface complexation and ligand-exchange reactions involving surface hydroxyl groups [25], which may contribute to NOR retention in these urban river sediments. Taking sediment S7 as a representative example, Fourier transform infrared (FTIR) spectra of the NOR-loaded sediment exhibited more pronounced absorption features at 1633 and 1400 cm^−1^ (Figure 1e,f), indicating that the carboxylate moiety may be involved in interactions between NOR and sediment surfaces [26]. A negative correlation was observed between Q_m_ and TP contents (r = −0.68). Although this trend might suggest the influence of phosphate-associated minerals or organic phases, which could occupy reactive surface sites, thereby reducing the availability of adsorption sites for NOR [27], this interpretation must be considered preliminary given the small sample size.

The Langmuir constant b, which reflects the affinity between NOR and the sediment surface, was positively correlated with LOI (r = 0.64) and C/N ratios (r = 0.71). LOI is widely used as a proxy for sediment organic matter content, whereas elevated C/N ratios generally indicate a larger proportion of terrestrial, less-decomposed organic matter enriched in lignin-derived aromatic moieties and oxygen-containing functional groups [28,29]. These positive trends may indicate that sediment organic matter predominantly governs the affinity of NOR adsorption rather than the number of available adsorption sites. Furthermore, b showed a strong negative correlation with pH_aver_ (r = −0.79, *p* < 0.05), and the Freundlich capacity parameter (*K*_f_) exhibited an even stronger negative correlation with pH_aver_ (r = −0.93, *p* < 0.05). These results suggest that lower equilibrium pH strongly enhances both the affinity and overall capacity of NOR adsorption, likely due to the increased proportion of cationic NOR species and more favorable electrostatic interactions with the sediment surfaces [25]. It should be noted that several sediment physicochemical parameters showed strong intercorrelations. Such collinearity makes it difficult to attribute NOR adsorption to a single sediment property, and the observed adsorption variability is more likely governed by the combined effects of multiple factors. Overall, the isotherm and exploratory correlation results suggest that NOR adsorption on urban river sediments is controlled by finite adsorption sites, surface heterogeneity, and equilibrium pH.

### 2.2. Site Energy Distribution of NOR Adsorption

**Figure 2:**
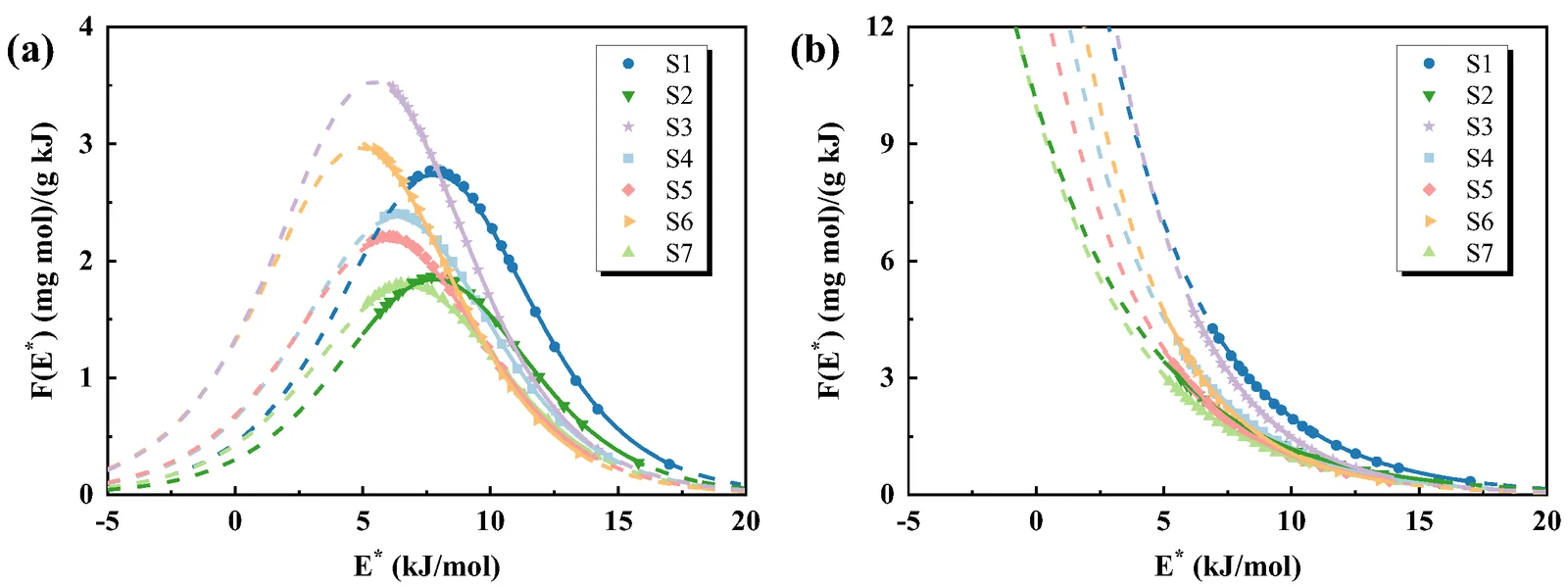
Site energy distribution curves of NOR adsorption on sediments derived from the (**a**) Langmuir and (**b**) Freundlich models. Experimental conditions: sediment dosage = 3 g/L; [NOR]_0_ = 10–200 μM.

To further resolve the energetic heterogeneity underlying NOR adsorption, adsorption site energy distribution analysis was performed based on the Langmuir-derived isotherm parameters. The solubility of NOR at pH 7 in pure water was determined to be 238 mg/L, which was used to calculate the site energy distribution functions. The resulting energy distribution curves for NOR adsorption on the collected urban river sediments are shown in Figure 2a, and the corresponding characteristic parameters are summarized in Table 2.

The adsorption of NOR on the urban river sediments exhibited approximately quasi-Gaussian, single-peaked energy distribution curves. The distribution curve reached its maximum F(E_0_^*^) at the characteristic adsorption energy E_0_^*^. The region to the left of E_0_^*^ represents relatively low-energy adsorption sites, whereas the region to the right corresponds to relatively high-energy sites. The decrease in F(E^*^) on both sides of E_0_^*^ indicates that extremely low- or high-energy sites were less abundant and contributed less to the overall adsorption process. Clear differences in both peak position and peak intensity were observed among the sediments, suggesting sediment-dependent variations in adsorption site strength and site abundance. S1 and S2 exhibited the highest E_0_^*^ values of 7.823 and 7.796 kJ/mol, respectively, together with high average adsorption energies E_m_ of 8.155 and 8.133 kJ/mol. However, their Langmuir adsorption capacities were not the highest, suggesting that stronger characteristic adsorption energy alone was insufficient to determine overall NOR retention. In contrast, S3 and S6 exhibited relatively high peak frequencies, with F(E_0_^*^) values of 3.578 and 3.008 (mg mol)/(g kJ), respectively, despite their lower E_0_^*^ values of 5.389 and 4.844 kJ/mol. Moreover, these two sediments showed relatively high adsorption capacities in the isotherm analysis, indicating that the adsorption capacity was more closely associated with the abundance of effective adsorption sites than with the absolute strength of site energy.

The distribution heterogeneity σ reflects the differences in the width of energy distributions among the sediments. S1 and S2 showed relatively large σ values of 3.816 and 3.814, respectively, indicating broader energy distributions and stronger energetic heterogeneity. S7 also exhibited a relatively broad distribution, with a σ value of 3.734. By comparison, S3 and S6 exhibited lower σ values of 3.589 and 3.521, respectively, suggesting more concentrated energy distributions. Thus, a broader energy distribution did not necessarily correspond to greater adsorption capacity. Larger σ values generally suggest the coexistence of multiple types of adsorption sites with different binding strengths. It has been reported that organic matter, clay minerals, and metal oxides in natural sediments can contribute diverse adsorption domains through hydrophobic interactions, hydrogen bonding, π–π interactions, electrostatic attraction, and surface complexation [30,31]. As a result, sediments enriched in these reactive components often exhibit broader energy distributions and greater energetic heterogeneity. In contrast, sediments with lower organic matter contents tend to possess more homogeneous surface properties, resulting in narrower energy distributions [31].

As a methodological cross-check, site energy distributions were also derived from the Freundlich parameters. Unlike the Langmuir-based curves, the Freundlich-derived distributions are monotonic exponential functions without any interior maximum (Figure 2b). Because the Freundlich model lacks a saturation capacity, no characteristic energy (E_0_^*^) or peak frequency F(E_0_^*^) can be defined; the apparent boundary values are determined by the chosen reference concentration (C_s_) rather than intrinsic surface properties. Furthermore, the integrated parameters (E_m_ and σ) are mathematically constrained by the single exponent n. This limitation arises from the mathematical structure of the Freundlich equation itself, rather than from the experimental data. Therefore, the Langmuir-based analysis was retained for all subsequent site energy accounting, as it uniquely provides an interpretable peak position (E_0_^*^), site abundance (F(E^*^)), and mutually independent energy descriptors.

Overall, site energy distribution analysis provides mechanistic information beyond conventional isotherm fitting. These results show that differences in NOR adsorption among the sediments were governed not only by adsorption energy intensity but also by the abundance and accessibility of favorable adsorption domains. This distinction is particularly important for natural sediments, where similar macroscopic adsorption behaviors may arise from different underlying energetic structures.

### 2.3. Effects of Coexisting Ions on NOR Adsorption and Site Energy Distribution

**Figure 3:**
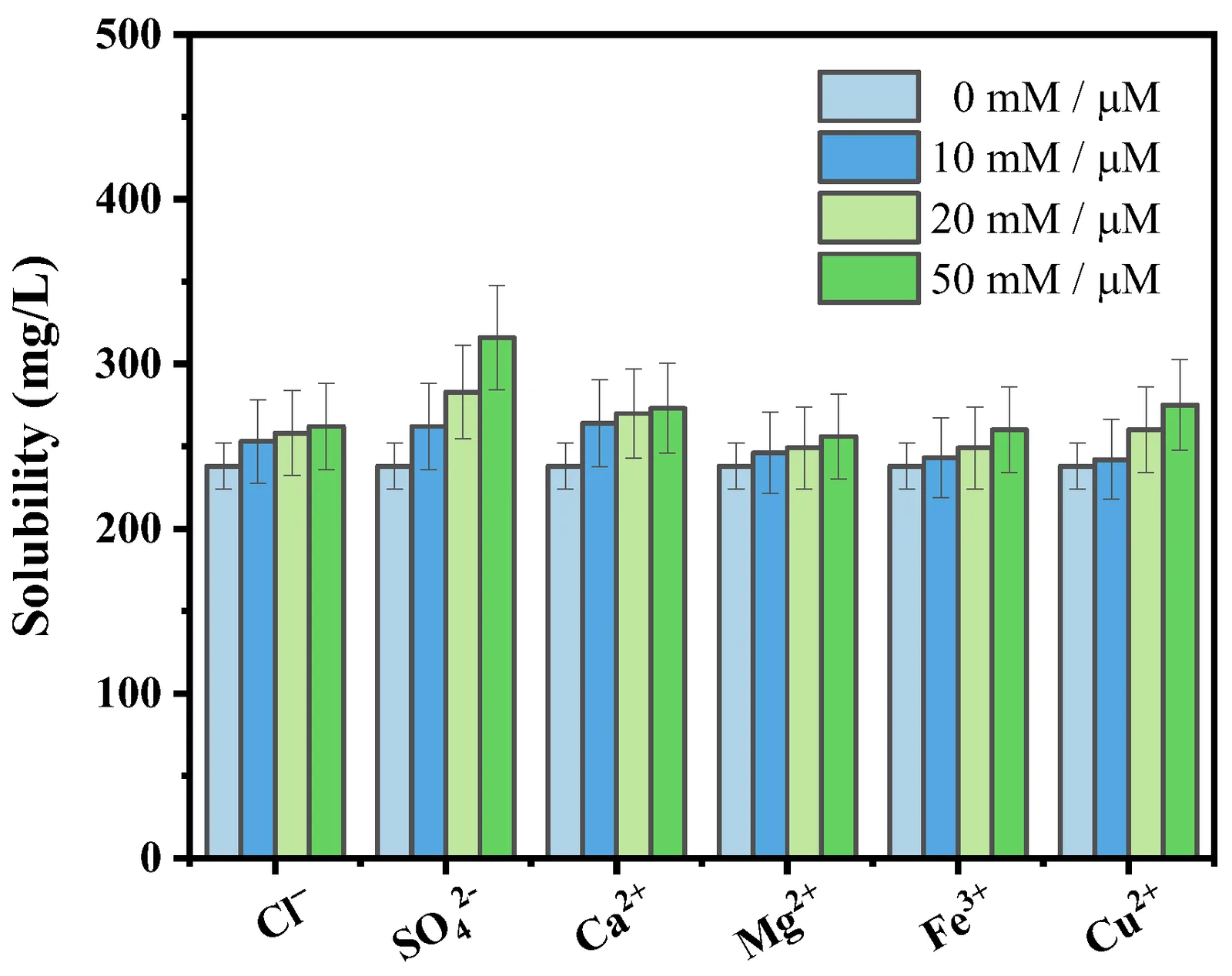
Solubility of NOR under coexisting ion conditions with Cl^−^ and SO_4_^2−^ at 0–50 mM and Ca^2+^, Mg^2+^, Fe^3+^, and Cu^2+^ at 0–50 μM.

Natural aquatic systems contain a variety of dissolved inorganic ions that can substantially affect antibiotic adsorption [10]. These effects are particularly pronounced for fluoroquinolones like NOR, whose zwitterionic nature and multiple functional groups make their adsorption highly sensitive to solution chemistry. To elucidate the role of coexisting ions, adsorption isotherms and adsorption site energy distributions of NOR were investigated in the presence of three representative ion groups: (i) common electrolytes (Cl^−^ and SO_4_^2−^) at environmentally relevant millimolar concentrations (0–50 mM); (ii) divalent alkaline earth metal cations (Mg^2+^ and Ca^2+^) at micromolar concentrations (0–50 μM); and (iii) strongly complexing metal cations (Fe^3+^ and Cu^2+^) at comparable micromolar levels. By combining conventional isotherm analysis with SEDT, the following sections investigate how different ion types and concentrations regulate NOR adsorption capacity and site energy distribution characteristics. The solubility of NOR under each ion condition was determined (Figure 3), as required for the site energy distribution calculation.

#### 2.3.1. Effects of Common Electrolytes

**Figure 4:**
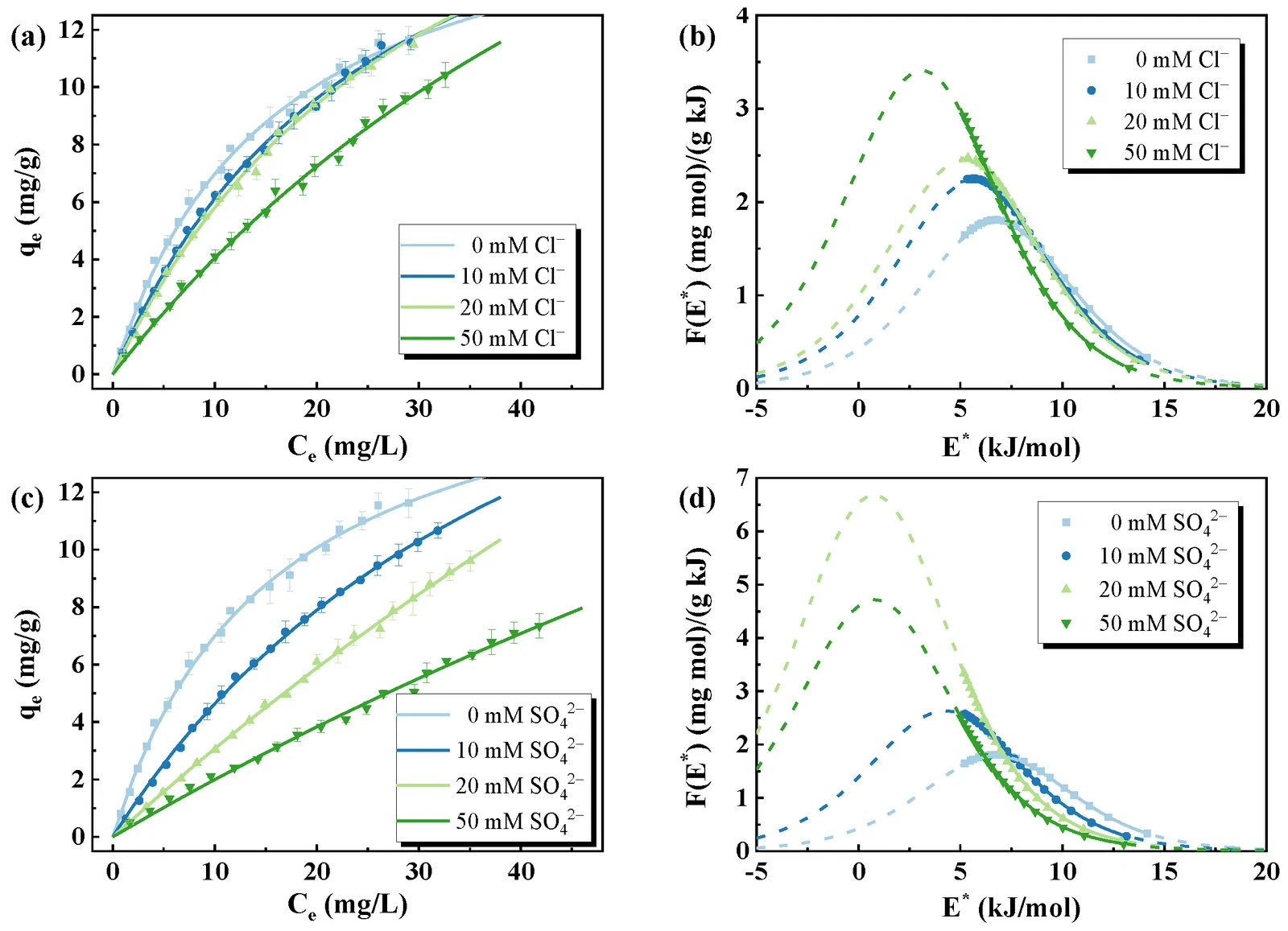
Effects of common electrolytes on NOR adsorption and site energy distributions: (**a**,**c**) Adsorption isotherms and (**b**,**d**) site energy distribution curves in the presence of (**a**,**b**) Cl^−^ and (**c**,**d**) SO_4_^2−^, respectively. Experimental conditions: sediment dosage = 3 g/L; [NOR]_0_ = 10–200 μM; [Cl^−^] = 0–50 mM; [SO_4_^2−^] = 0–50 mM.

As shown in Figure 4a,c, the q_e_ of NOR on the urban river sediments consistently decreased with increasing electrolyte concentrations for both NaCl and Na_2_SO_4_, indicative of weakened adsorbate-adsorbent affinity at higher ionic strengths. This effect was more pronounced for Na_2_SO_4_ than NaCl at the same molar concentration, as reflected by the greater decrease in adsorption amount over the entire equilibrium concentration range. The different effects of NaCl and Na_2_SO_4_ can be largely explained by their ionic strength. According to the ionic strength definition shown in Equation (1), where I is the ionic strength, c_i_ denotes the molar concentration of each ion, and z_i_ represents the corresponding ionic charge number, NaCl solutions at 10, 20, and 50 mM correspond to ionic strengths of 10, 20, and 50 mM, respectively, whereas Na_2_SO_4_ yields substantially higher ionic strengths of 30, 60, and 150 mM at the same molar levels. The greater ionic strength of sulfate-containing solutions likely enhances electrostatic screening and compresses the electrical double layer more effectively, thereby suppressing NOR adsorption to a greater extent. The site energy distribution analysis further confirmed the inhibitory effect of increasing electrolyte concentrations (Figure 4b,d). For both the NaCl and Na_2_SO_4_ systems, the energy distribution curves shifted progressively toward lower E^*^ values with increasing salt concentrations, accompanied by a reduced contribution from high-energy adsorption sites. This trend indicates that elevated electrolyte concentrations modified the energetic contribution of adsorption sites. In particular, Na_2_SO_4_ caused a stronger redistribution of adsorption site energies than NaCl, consistent with its greater inhibitory effect on adsorption capacity.

(1) $$I=\frac{1}{2}\sum c_{i}z_{i}^{2}$$

The observed suppression of NOR adsorption with increasing ionic strength conforms to some reports on fluoroquinolone antibiotics and other charged organic contaminants. Cao et al. [32] reported that NOR adsorption on marine sediments was negatively correlated with salinity, indicating that increased salinity weakened the partitioning of NOR from the aqueous phase to sediments. Similar behavior was observed for ofloxacin on marine sediments, where the adsorption capacity decreased with increasing salinity, suggesting that high-salinity conditions can reduce the retention of fluoroquinolones by sediments [33]. Besides the electrostatic screening effect as mentioned above, the competition between electrolytes and ionic NOR species for binding sites also contributes to the decrease in adsorption [34,35]. Moreover, the slight increase in NOR solubility under sulfate-rich conditions may further lower the partitioning affinity for NOR between the aqueous and sediment phases, thereby reinforcing the apparent inhibition of adsorption [34]. These mechanisms collectively explain the decreasing NOR adsorption and the observed migration of adsorption site energy distributions toward lower-energy regions with increasing NaCl and Na_2_SO_4_ concentrations.

#### 2.3.2. Effects of Divalent Alkaline Earth Metal Cations

**Figure 5:**
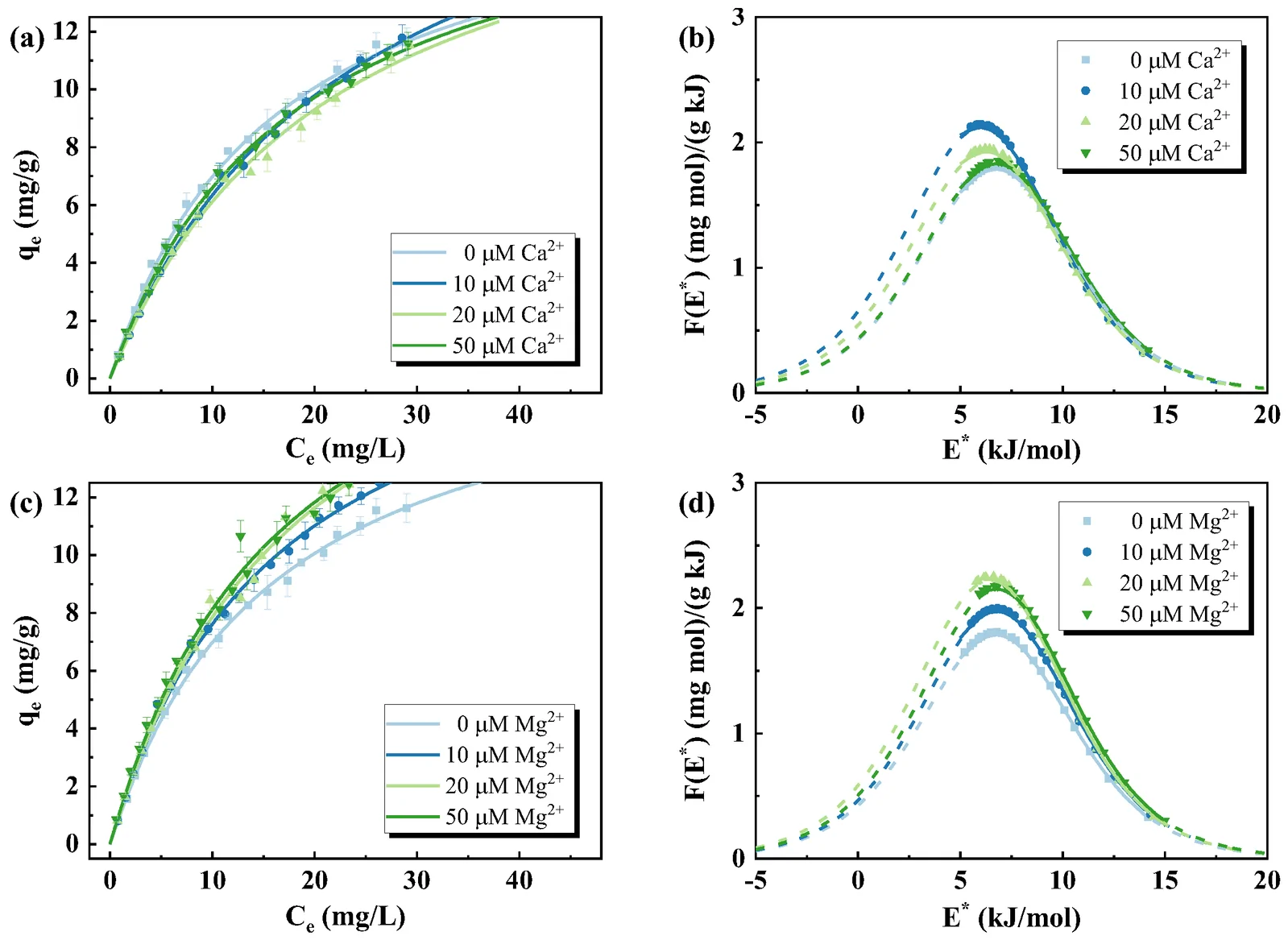
Effects of divalent alkaline earth metal cations on NOR adsorption and site energy distributions: (**a**,**c**) Adsorption isotherms and (**b**,**d**) site energy distribution curves in the presence of (**a**,**b**) Ca^2+^ and (**c**,**d**) Mg^2+^, respectively. Experimental conditions: sediment dosage = 3 g/L; [NOR]_0_ = 10–200 μM; [Ca^2+^] = 0–50 μM; [Mg^2+^] = 0–50 μM.

As shown in Figure 5a,c, Ca^2+^ had little effect on NOR adsorption, with the isotherms at different concentrations largely overlapping. Mg^2+^ slightly enhanced adsorption, especially at higher equilibrium concentrations, but did not change the overall isotherm shape. These results indicate that neither Ca^2+^ nor Mg^2+^ fundamentally altered the adsorption mode of NOR within 0–50 μM. The site energy distribution curves provided additional support for these weak effects (Figure 5b,d). In the Ca^2+^ system, the slight changes in peak frequency F(E^*^) were observed, with no obvious shift in the dominant energy region, indicating negligible changes in the adsorption energy and heterogeneity. In the Mg^2+^ system, the peak position remained nearly unchanged, while F(E^*^) increased, suggesting the enhanced utilization of existing adsorption sites rather than the formation of new high-energy sites or changes in the intrinsic energy characteristics.

The weak effects of Ca^2+^ and Mg^2+^ in this study can be largely attributed to their low concentration range. At 0–50 μM, the ionic strength introduced by these divalent cations was much lower than that generated by millimolar electrolytes in Section 2.3.1, and the electrostatic screening and electrical double layer compression were therefore limited. From a molecular perspective, fluoroquinolones possess keto and carboxyl groups that can complex metal ions. Direct complexation between NOR and Mg^2+^/Ca^2+^ has been demonstrated by crystal structure analysis, confirming the chemical basis for metal-NOR complex formation [36]. However, the adsorption consequence of such complexation is not fixed: metal-NOR complexation may either promote surface association through cation bridging or enhance the aqueous solubility of NOR, depending on ion concentrations, NOR speciation, surface charge, and the availability of mineral binding sites. In the present study, Ca^2+^ caused almost no change in the isotherms or energy distributions, and Mg^2+^ induced only a slight increase in F(E^*^) without shifting the characteristic energy region. The phenomenon of Ca^2+^ and Mg^2+^ effects should therefore be interpreted as concentration-limited modulation, rather than as evidence for substantial restructuring of NOR adsorption energetics mediated by alkaline earth metals.

#### 2.3.3. Effects of Strongly Complexing Metal Cations

**Figure 6:**
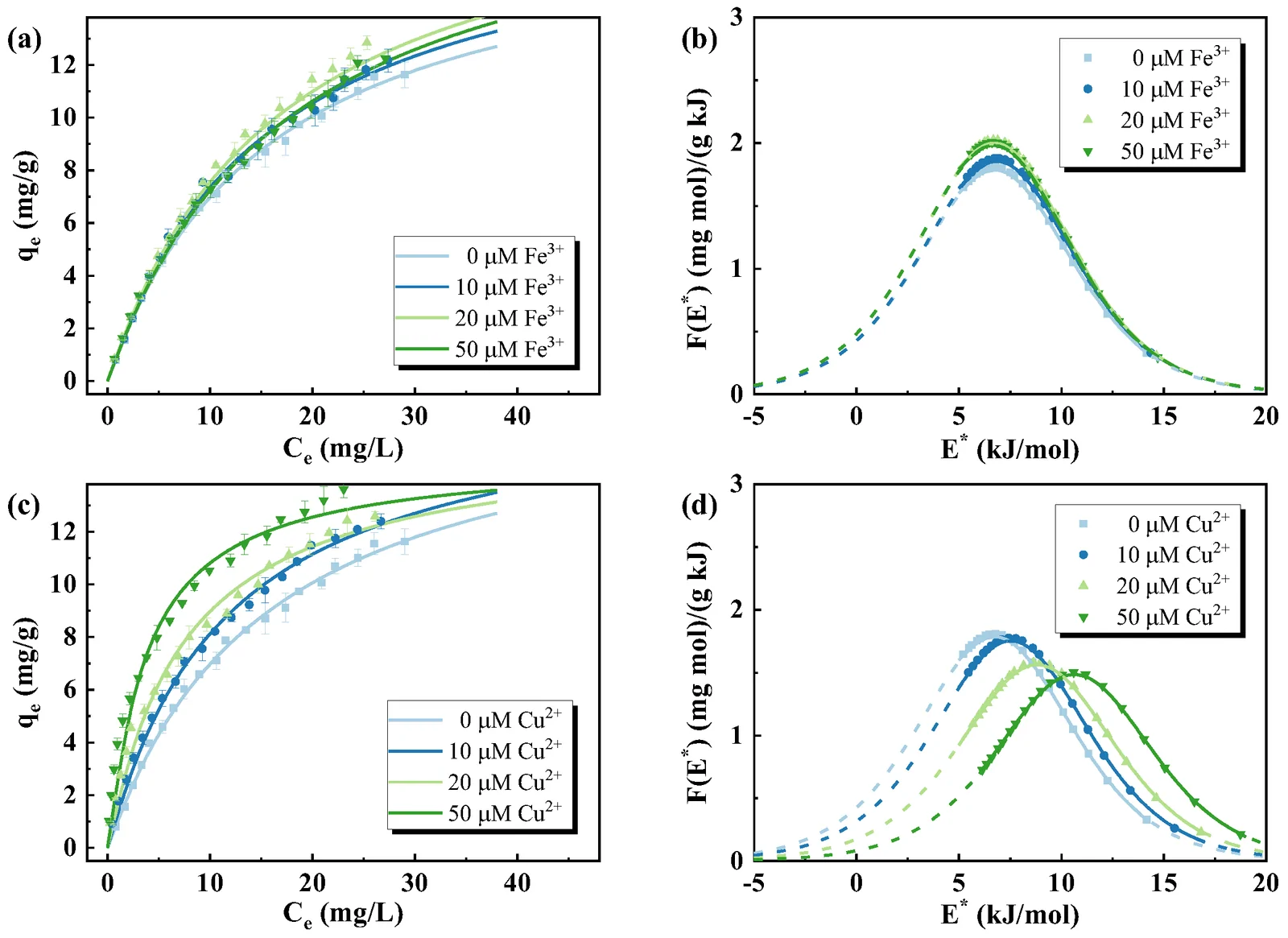
Effects of strongly complexing metal cations on NOR adsorption and site energy distributions: (**a**,**c**) Adsorption isotherms and (**b**,**d**) site energy distribution curves in the presence of (**a**,**b**) Fe^3+^ and (**c**,**d**) Cu^2+^, respectively. Experimental conditions: sediment dosage = 3 g/L; [NOR]_0_ = 10–200 μM; [Fe^3+^] = 0–50 μM; [Cu^2^] = 0–50 μM.

The effects of strongly complexing metal cations on NOR adsorption were investigated using Fe^3+^ and Cu^2+^ at concentrations of 0–50 μM. As shown in Figure 6a,c, Fe^3+^ caused only a slight enhancement of NOR adsorption, despite isotherms largely overlapping those of the Fe^3+^-free system at low concentrations, indicating no substantial alteration of the overall adsorption mode. In contrast, Cu^2+^ produced a much more pronounced promotion effect. With increasing Cu^2+^ concentration, the adsorption isotherms shifted upward, particularly at lower equilibrium concentrations, suggesting enhanced affinity between NOR and the sediment surface. The adsorption site energy distribution results further demonstrate the distinct roles of Fe^3+^ and Cu^2+^ (Figure 6b,d). For Fe^3+^, the energy distribution curves showed only slight changes, with a minor increase in F(E^*^) and no obvious shift in the dominant energy region. This indicates that Fe^3+^ mainly increased the utilization of existing adsorption sites to a limited extent, without substantially changing the intrinsic adsorption energy distribution. Cu^2+^ caused a clear redistribution of adsorption site energies. As Cu^2+^ concentration increased, the distribution curves shifted toward higher E^*^ values, and the dominant adsorption region moved from approximately 6–8 kJ/mol to a higher-energy range. Although the peak frequency did not increase, the contribution of the high-energy region became more prominent, suggesting that Cu^2+^ strengthened the utilization affinity of stronger adsorption sites.

**Figure 7:**
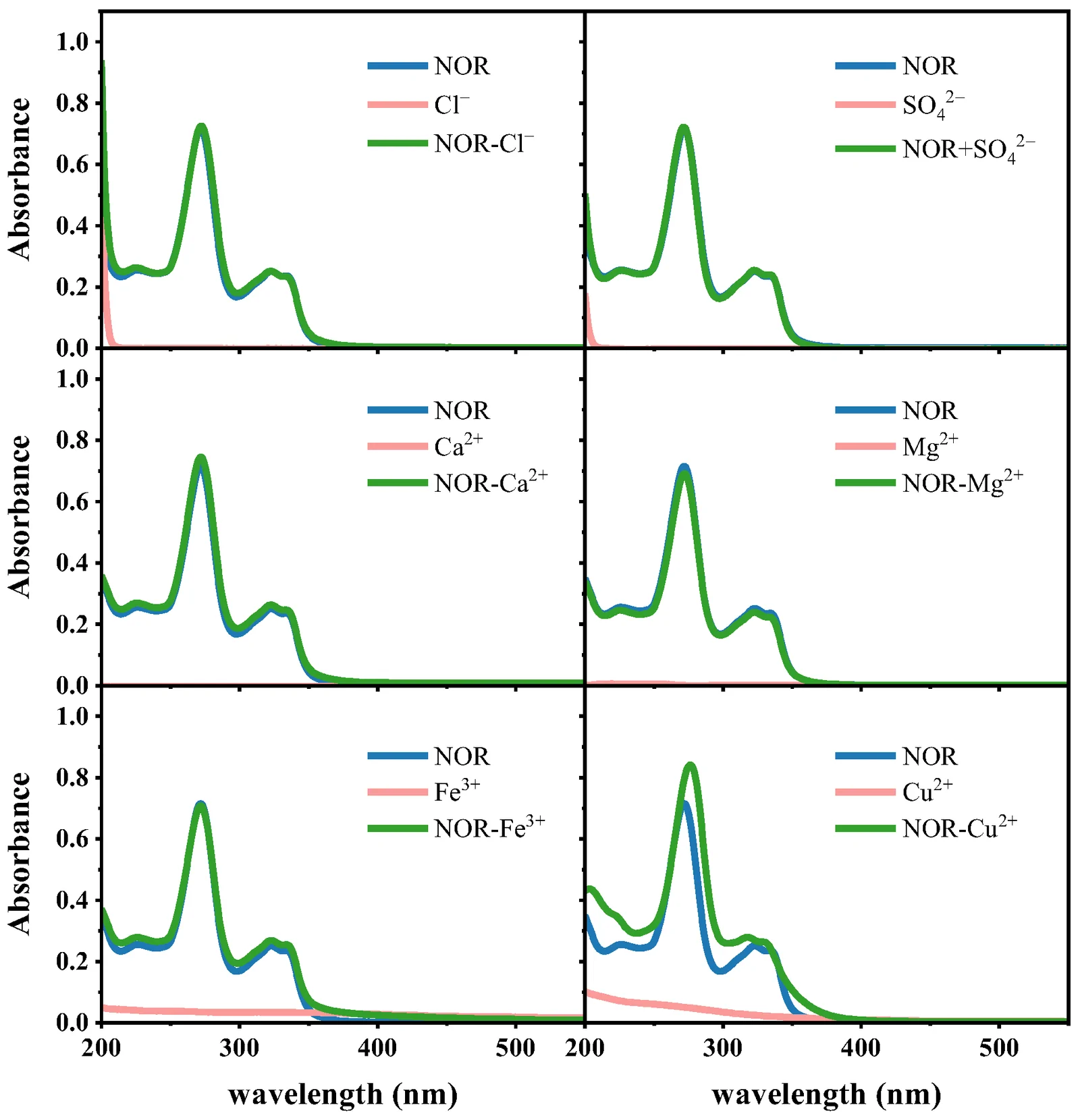
UV-Vis absorption spectra of NOR alone, ions alone, and NOR-ion mixtures. Experimental conditions: [NOR] = 20 μM; [Cl^−^] = [SO_4_^2−^] = 50 mM; [Ca^2+^] = [Mg^2+^] = [Fe^3+^] = [Cu^2+^] = 50 μM.

The stronger effect of Cu^2+^ is attributed to its high complexation affinity with NOR, which contains carboxyl and carbonyl groups that readily complex Cu^2+^ to form stable complexes [37,38]. Such complexes may enhance adsorption through cation bridging or ternary surface complex formation, in which Cu^2+^ acts as a bridge between NOR and negatively charged sediment or mineral surfaces [9]. UV-Vis spectra confirmed the ion-specific interaction between NOR and Cu^2+^ (Figure 7). The spectra of NOR mixed with Cl^−^, SO_4_^2−^, Ca^2+^, Mg^2+^, and Fe^3+^ showed little change compared with NOR alone, whereas Cu^2+^ induced a clear change in the NOR absorption band, reflecting the stronger complexation between Cu^2+^ and NOR. Previous studies also showed that coexisting Cu^2+^ enhances fluoroquinolone adsorption on clays or minerals through coadsorption and surface complexation, with Cu^2+^–ciprofloxacin complexes identified on montmorillonite and kaolinite surfaces [26,37].

By comparison, Fe^3+^ possesses a high charge density and strong complexation potential, but its behavior under near-neutral conditions is complicated by rapid hydrolysis and speciation changes. Aqueous Fe(III) can form hydrolyzed species such as FeOH^2+^, Fe(OH)_2_^+^, Fe(OH)_3_^0^, and Fe(OH)_4_^−^ [39], which can reduce the concentration of freely available Fe^3+^ for direct NOR complexation. Meanwhile, hydrolyzed Fe species or newly formed Fe hydroxide colloids may introduce additional adsorption domains [40], but they may also compete with NOR for sediment surface sites or mask pre-existing high-affinity sites. Therefore, Fe^3+^ may exert mixed effects involving complexation, surface association with Fe hydroxyl species, and partial site blocking, which could explain why Fe^3+^ only slightly enhanced NOR adsorption despite its high charge and complexation ability.

## 3. Materials and Methods

### 3.1. Chemicals and Materials

NOR (98%) was purchased from Aladdin Industrial Co., Ltd. (Shanghai, China). The stock solution of NOR was prepared in ultrapure water and stored in the dark at 4 °C. The working solutions were freshly diluted prior to each experiment. The inorganic salts, including NaCl, Na_2_SO_4_, CaCl_2_, MgCl_2_·6H_2_O, FeCl_3_, and CuCl_2_·2H_2_O, were purchased from Sinopharm Chemical Reagent Co., Ltd. (Shanghai, China), and the purity levels were of analytical grade or higher. Ultrapure water with 18.2 MΩ cm resistivity was used throughout the experiments.

### 3.2. Sediment Collection and Characterization

**Figure 8:**
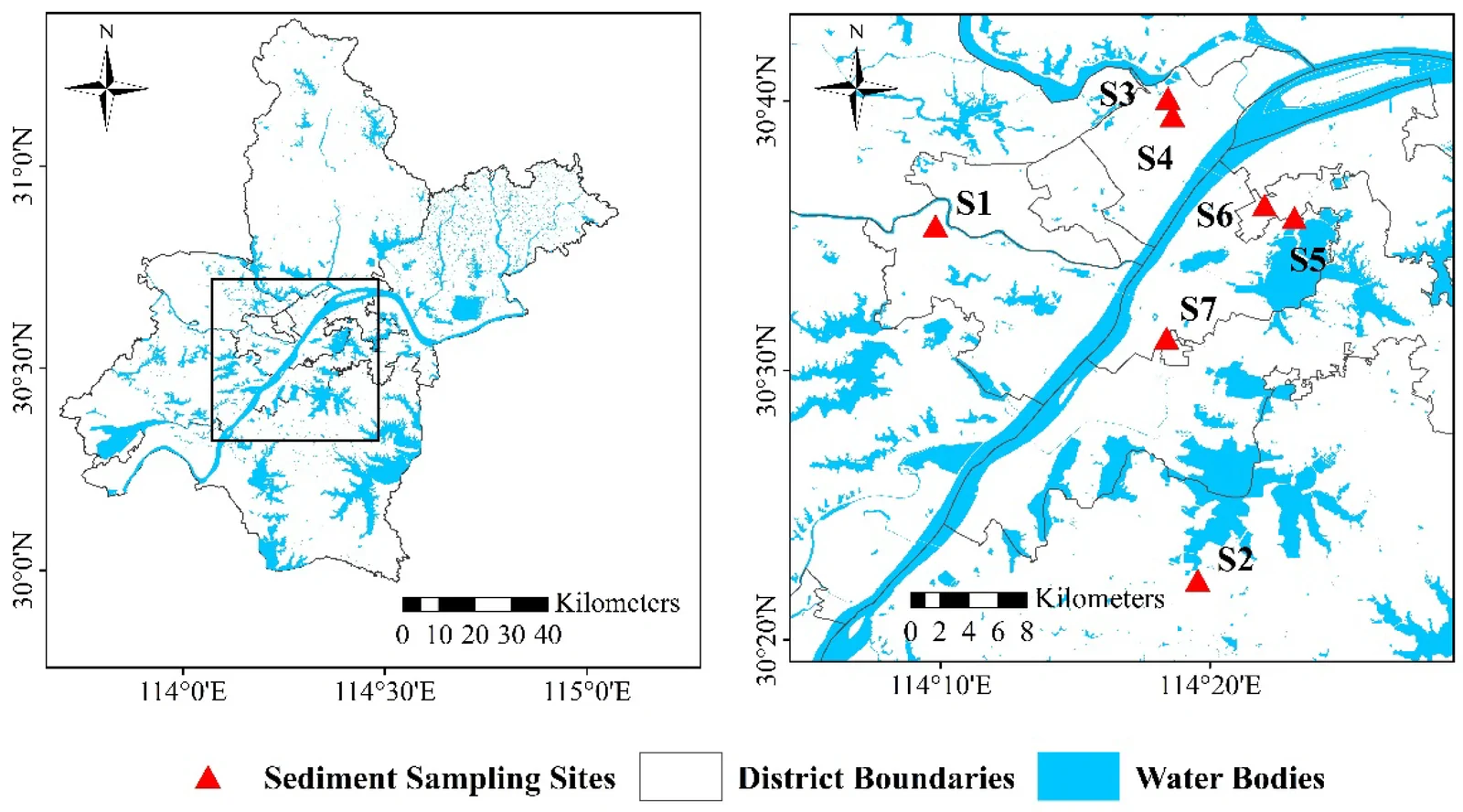
Map showing the locations of the sediment sampling sites.

Wuhan, located in the middle reaches of the Yangtze River, is renowned for its numerous rivers and lakes. The rapid urbanization and high population growth have led to the widespread occurrence of antibiotics in Wuhan’s aquatic environments, posing significant challenges to water environmental management. Sediment samples were collected using a grab sampler from seven representative urban rivers across the city in August 2020, reflecting the spatial variability of urban riverine environments (Figure 8). At each sampling site, a single grab sample of surface sediment (0–10 cm) was collected, and approximately one full grab was taken as the bulk sample for each site. The samples were sealed in clean polyethylene bags and transferred immediately to the lab. The collected sediments were air-dried at room temperature, then gently ground and sieved using a 100-mesh sieve prior to use.

**Figure 9:**
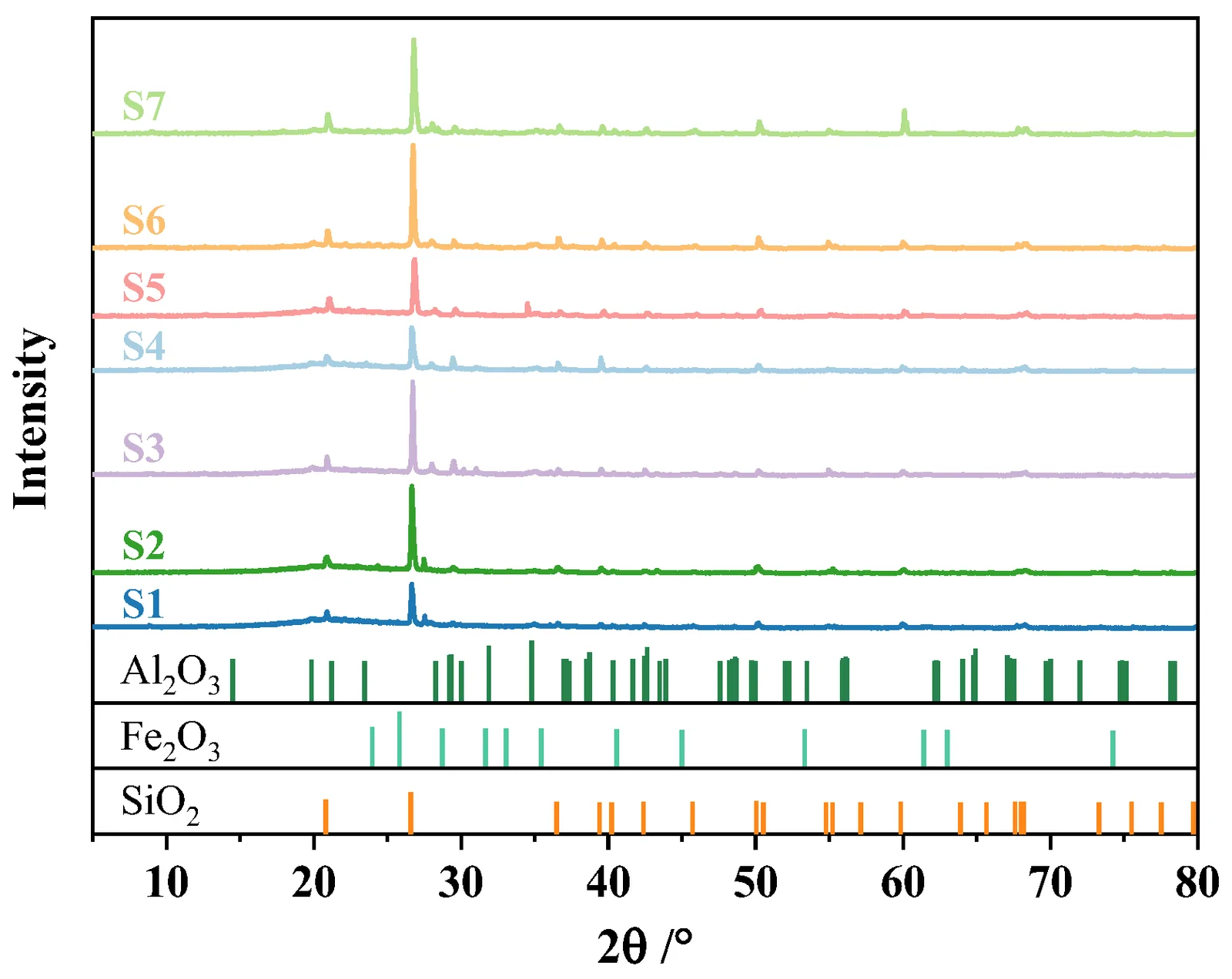
XRD patterns of the sediment samples. The standard reference patterns for SiO_2_, Fe_2_O_3_, and Al_2_O_3_ are provided at the bottom for comparison.

Basic physicochemical characteristics of the sediments were determined following standard procedures. Mineral phases were identified by powder X-ray diffraction (XRD, Rigaku MiniFlex 600, Tokyo, Japan). The XRD patterns of the seven sediments were broadly similar, and distinct diffraction peaks attributable to quartz were observed (Figure 9). The presence of poorly crystalline or amorphous mineral components cannot be excluded based on the XRD results; consequently, the total Fe oxide content (expressed as Fe_2_O_3_ equivalent) was quantified using a handheld X-ray fluorescence (XRF) spectrometer (Explorer 9000, Innov-X Systems, Waltham, MA, USA). SA was obtained using a surface area and porosity analyzer (V-Sorb 2800P, Gold APP Instruments, Beijing, China). ZP and PD were measured using a laser particle size analyzer (Zetasizer Nano ZEN3700, Malvern Panalytical, Malvern, UK). LOI was measured by igniting samples at 550 °C for 5.5 h in a muffle furnace. The C/N of sediments was determined using an elemental analyzer (vario MACRO cube, Elementar, Hanau, Germany). TP content was analyzed via the alkali fusion–Mo-Sb anti spectrophotometric method. The summarized physicochemical properties of the sediments are given in Table 3.

### 3.3. Adsorption Experiments

Batch adsorption experiments were conducted to investigate the adsorption behaviors of NOR on the urban river sediments. 0.03 g of sediment was added to a series of centrifuge tubes containing 10 mL of NOR solutions with initial concentrations ranging from 10 to 200 μM at 10 μM intervals. Although NOR is typically detected in natural river waters at environmentally relevant concentrations (ng/L to μg/L levels) [41], much higher concentrations are often required in batch experiments to reach the saturation plateau for reliable isotherm and site energy distribution modeling. All experiments were initiated at an initial pH of 7, which was adjusted using dilute HCl or NaOH solutions. The mixtures were agitated at room temperature for 24 h to ensure adsorption equilibrium, as determined by preliminary kinetic experiments. After equilibration, the suspensions were filtered through 0.22 μm polyethersulfone filters for subsequent concentration analysis. All batch adsorption experiments were performed at least twice.

To examine the effects of coexisting ions, a representative sediment (S7) was selected, and adsorption experiments were performed in the presence of varying concentrations of inorganic ions, including common electrolytes (Cl^−^ and SO_4_^2−^), divalent alkaline earth metal cations (Mg^2+^ and Ca^2+^), and strongly complexing metal cations (Fe^3+^ and Cu^2+^). The concentration ranges of the electrolytes were 0–50 mM, and those of the metal cations were 0–50 μM. The pH was adjusted to 7 after adding the ions. All other experimental conditions were kept identical to those in the single NOR adsorption experiments to facilitate direct comparison.

### 3.4. Analytical Methods

The residual concentrations of NOR after adsorption were determined using high-performance liquid chromatography (HPLC) equipped with a 20ADVP pump and a DAD-20AVP detector (Shimadzu, Kyoto, Japan) [10]. Chromatographic separation was achieved on a C18 column (5 μm, 250 mm × 4.6 mm; Supelco Discovery, Bellefonte, PA, USA) maintained at 40 °C, with a flow rate of 1.0 mL/min. The mobile phase consisted of 0.1% formic acid solution and 18% acetonitrile. The injection volume was 100 μL, and NOR was detected at a wavelength of 283 nm.

The solubility of NOR was determined for site energy distribution calculations. Experiments were conducted in ultrapure water at pH 7, without or with different concentrations of coexisting ions. Excess NOR powder was added to each solution to form a saturated suspension with undissolved solids. The pH was adjusted and maintained at 7 ± 0.05 during the dissolution. The suspensions were filtered and diluted to determine the dissolved NOR concentrations, which served as the solubility under corresponding conditions.

UV-Vis absorption spectra of NOR with and without coexisting ions were measured on a UV-3600 (Shimadzu, Kyoto, Japan) spectrophotometer to explore ion-NOR interactions; the individual ion solutions were also scanned separately to provide background references. FTIR spectra of the sediments (before and after adsorption) were recorded on a Nicolet 5700 FT-IR spectrometer (Thermo Electron Corporation, Waltham, MA, USA). Dried samples were mixed with KBr and pressed into pellets. Spectra were normalized to the 850–450 cm^−1^ mineral matrix band to eliminate loading differences.

### 3.5. Data Analysis

The q_e_ values (mg/g) were calculated based on the mass balance between initial and equilibrium NOR concentrations (C_0_ and C_e_, mg/L) according to Equation (2), where V (L) is the solution volume, and m (g) is the mass of sediment used for the adsorption. The Langmuir model and Freundlich model were employed to characterize the adsorption behaviors of NOR and are expressed by Equations (3) and (4). In Equation (4), *K*_f_ is the Freundlich capacity parameter related to the maximum adsorption capacity, and *n* is the heterogeneity index.

(2) $$q_{e}=\frac{(C_{0}-C_{e})\times V}{m}$$

(3) $$q_{e}=\frac{Q_{m}bC_{e}}{1+bC_{e}}$$

(4) $$q_{e}=K_{f}{C_{e}}^{n}$$

To further characterize the heterogeneity of adsorption sites, adsorption site energy distribution analysis was performed by transforming isotherm data into energy distribution functions. According to the adsorption SEDT, the adsorbent surface consists of a population of adsorption sites with different binding energies [42]. At a given C_e_, adsorption occurs only on sites with binding energies exceeding a threshold energy (E^*^, kJ/mol) [43]. The relationship between C_e_ and E^*^ is given by Equation (5), where C_s_ (mg/L) is the solubility of the adsorbate, R is the universal gas constant (8.314 J/(mol K)), and T (K) is the absolute temperature. Higher E^*^ values indicate stronger adsorption, whereas E^*^ approaches zero as C_e_ approaches C_s_, corresponding to weak adsorption conditions [42]. Combining Equations (3) and (5), q_e_ can be expressed as a function of E^*^ (q_e_(E^*^)). The site energy distribution function F(E^*^) based on the Langmuir isotherm model was then obtained by differentiating q_e_ with respect to E^*^, as shown in Equation (6). Here, F(E^*^) represents the frequency distribution of adsorption sites with energy E^*^, reflecting the overall energetic heterogeneity of the adsorbent surface. The site energy distribution based on the Freundlich model was also derived using an analogous approach and calculated according to Equation (7).

(5) $$C_{e}=C_{s}\exp(\frac{-E^{*}}{RT})$$

(6) $$F\left(E^{*}\right)=-\frac{dq_{e}(E^{*})}{dE^{*}}=\frac{Q_{m}bC_{s}}{RT}\exp\left(\frac{-E^{*}}{RT}\right){\left[1+bC_{s}\exp\left(\frac{-E^{*}}{RT}\right)\right]}^{-2}$$

(7) $$F\left(E^{*}\right)=\frac{K_{f}n{C_{s}}^{n}}{RT}\exp\left(\frac{-nE^{*}}{RT}\right)$$

Two integrated parameters, E_m_ and σ, were used to quantitatively characterize the overall features of the energy distributions. E_m_ reflects the average interaction strength between NOR and the sediment surface, whereas σ describes the breadth of the energy distribution and thus the degree of surface energetic heterogeneity. The calculation methods are given in Equations (8) and (9), where E_min_^*^ (kJ/mol) and E_max_^*^ (kJ/mol) correspond to the intersections of the low- and high-energy regions of the distribution curve with the energy axis, respectively [44].

(8) $$E_{m}=\frac{\int_{{E_{min}}^{*}}^{{E_{max}}^{*}}E^{*}\times F\left(E^{*}\right)dE^{*}}{\int_{{E_{min}}^{*}}^{{E_{max}}^{*}}F\left(E^{*}\right)dE^{*}}$$

(9) $$\sigma =\sqrt{\frac{\int_{{E_{min}}^{*}}^{{E_{max}}^{*}}{E^{*}}^{2}\times F\left(E^{*}\right)dE^{*}}{\int_{{E_{min}}^{*}}^{{E_{max}}^{*}}F\left(E^{*}\right)dE^{*}}-{E_{m}}^{2}}$$

## 4. Conclusions

This study investigated the NOR adsorption on urban river sediments and evaluated the influence of coexisting ions on adsorption behaviors and site energy distributions. NOR adsorption exhibited clear sediment-dependent variability and was well described by the Langmuir model, with maximum adsorption capacities Q_m_ ranging from 17.932 to 35.476 mg/g. Site energy distribution analysis demonstrated that higher adsorption capacities did not necessarily correspond to higher adsorption energies. Instead, NOR retention depended more on the abundance of effective adsorption sites. Regarding ionic effects, the common electrolytes, especially Na_2_SO_4_, inhibited adsorption and shifted site energy distributions toward lower-energy regions, mainly due to ionic strength effects. Ca^2+^ had little effect on NOR adsorption, while Mg^2+^ slightly enhanced adsorption. Fe^3+^ produced only a slight enhancement of NOR adsorption, whereas Cu^2+^ markedly promoted adsorption and shifted the site energy distributions toward higher-energy regions. UV-Vis spectra provided evidence for Cu^2+^-NOR complex formation, suggesting that specific metal-antibiotic complexation is responsible for the strong promotional effect of Cu^2+^. Overall, combining adsorption isotherms with site energy distribution analysis, this study offers a useful framework for understanding antibiotic adsorption on heterogeneous sediment surfaces and the effects of coexisting ions. These findings provide new insights into the environmental fate of antibiotics in urban river systems and have implications for assessing the ecological risks of antibiotic persistence.

## Figures and Tables

**Table 1:** Isotherm parameters of NOR adsorption on sediments.

Sediments	Langmuir				Freundlich			
	Q_m_ (mg/g)	b (L/mg)	R^2^	RMSE (mg/g)	*K_f_* ((mg/g)/(mg/L)^n^)	*n*	R^2^	RMSE (mg/g)
S1	27.477	0.099	0.995	0.335	3.190	0.623	0.986	0.580
S2	18.499	0.098	0.989	0.414	2.527	0.533	0.986	0.414
S3	35.476	0.037	0.998	0.172	1.651	0.750	0.991	0.408
S4	23.829	0.054	0.981	0.541	1.784	0.652	0.981	0.544
S5	21.915	0.047	0.998	0.172	1.507	0.648	0.986	0.419
S6	29.822	0.030	0.995	0.281	1.204	0.741	0.989	0.397
S7	17.932	0.064	0.997	0.183	1.738	0.582	0.985	0.421

**Table 2:** Parameters of adsorption site energy distributions of NOR on sediments.

Sediments	F(E_0_^*^) ((mg mol)/(g kJ))	E_0_^*^ (kJ/mol)	E_m_ (kJ/mol)	σ
S1	2.771	7.823	8.155	3.816
S2	1.866	7.796	8.133	3.814
S3	3.578	5.389	6.250	3.589
S4	2.403	6.335	6.960	3.694
S5	2.210	5.976	6.684	3.656
S6	3.008	4.844	5.863	3.521
S7	1.809	6.758	7.291	3.734

**Table 3:** Properties of the sediments used for adsorption experiments.

Sediments	SA (m^2^/g)	ZP (mv)	PD (μm)	LOI (%)	FeOx (%)	C/N	TP (g/kg)
S1	16.54	−17.23	1.08	8.93	6.49	12.51	1.655
S2	26.53	−17.87	1.00	10.94	4.89	7.07	2.665
S3	30.40	−17.60	0.88	8.71	6.47	6.04	1.774
S4	7.83	−16.37	1.04	6.77	5.52	10.44	1.678
S5	6.35	−18.80	1.65	8.68	5.78	10.02	2.334
S6	45.74	−17.27	0.91	6.43	5.58	2.01	1.678
S7	10.02	−16.93	0.53	7.54	6.45	6.13	2.625

## Data Availability

The data that support the findings of this study are available from the authors upon reasonable request.
